# IGFs in Dentin Formation and Regeneration: Progress and Remaining Challenges

**DOI:** 10.1155/2022/3737346

**Published:** 2022-04-06

**Authors:** Pengcheng He, Liwei Zheng, Xin Zhou

**Affiliations:** ^1^State Key Laboratory of Oral Diseases, Sichuan University, Chengdu, Sichuan Province 610041, China; ^2^National Clinical Research Center for Oral Diseases, Sichuan University, Chengdu, Sichuan Province 610041, China; ^3^West China School of Stomatology, Sichuan University, Chengdu, Sichuan Province 610041, China; ^4^Department of Pediatric Dentistry, West China Hospital of Stomatology, Sichuan University, Chengdu, Sichuan Province 610041, China

## Abstract

Tertiary dentin results from the interplay between the host defense and dental injury or infection. Modern endodontics aiming vital pulp treatment take the tertiary dentin formation as the interim step, with the final goal of a physiological pulp-dentin like tissue regeneration. Dental pulp stem cells have been nominated for contributing to differentiating into odontoblast-like cells who are responsible for reparative dentin formation. Understanding the original dentin formation mechanism provides us a blueprint while exploring the reparative dentin formation mechanism builds bridge to bonafide pulp-dentin tissue regeneration. Among all the regulators, growth factors have long been revealed under the spotlight. The insulin-like growth factor (IGF) family has been implicated in critical events of inducing dentin formation, which is essential for pulp treatment. The expression of IGF family members including IGF1, IGF1R, IGF2, and IGF2R has been well characterized in dental papilla cells, dental pulp stem cells, and periodontal ligament cells. Recent studies indicated IGF binding to the receptors activated pathways, including MAPK pathway, and AKT pathway, orchestrated proliferation, and differentiation, and finally, contributed to dentin formation. This review summarizes the role of IGF family in dentin formation during tooth development and tertiary dentin formation during dentin-pulp repair and sheds light on key parts of research for future treatment improvements.

## 1. Introduction

Both trauma and caries can lead to dentin-pulp injuries. Pulp diseases or injuries are not fatal diseases, however, markedly affect the quality of life. Over the last decades, improvements in the performance of capping materials have substantially decreased the pulp loss associated with caries and dental trauma. Although dental pulp diseases are common, and capping materials are more advanced, our ability to preserve dental pulps and recover their functions remains limited and challenged.

When the dentin-pulp is impaired, the interaction between direct tissue injury, infection, inflammation, and the host defense responses determines the final outcomes [1]. While the mild injury can induce early inflammatory defense responses, contributing to odontoblast activation and reactionary dentin deposition, long-term and more severe injury takes the lead of outcome to be out-of-balance and away from dentin regeneration [2]. Tertiary dentin formation plays an important role in injury repairing and promoting pulp healing. Understanding the mechanism beneath will underpin and drive biologically based healing strategies ahead.

Growth factors (GFs) are a kind of proteinic bioactive agents who are essential to tissue growth and regulate cell fate [3]. GFs are usually stored in the extracellular matrix, where the proteases/enzymes often induce their cleavages. GFs are divided into different families, including translate growth factors (TGFs), bone morphogenetic proteins (BMPs), connective tissue growth factors (CTGFs), vascular endothelial growth factors (VEGFs), and insulin-like growth factors (IGFs) [4]. Growth factors can be secreted through autocrine, paracrine, or incretion and then bind to specific transmembrane receptors to trigger the subsequent bioreaction [5]. Each growth factor can only interact with one exclusive receptor among the others on the cells' superficies. The bioeffects induced by GF-receptor binding possess following features: (1) multitargeting: one singular growth factor can act on multiple target cells inducing multiple bioeffect; (2) overlapping: multiple growth factors can act on one target cell inducing similar bioeffect; (3) antagonism: one growth factor may trigger the bioeffect against another one; (4) synergism: one growth factor may trigger the bioeffect enhancing another one; (5) crosslinking: growth factors act synergistically or antagonistically with each other.

To achieve the injury repairing and promote pulp healing, multiple GFs reveal their essential roles in the formation of odontoblasts and odontoblast-like cells, subsequently leading to enhancement of dentin formation and tertiary dentin formation. Among these factors, the IGF family stands out in the spotlight and shows great potential for future bioactive pulp treatment.

## 2. Dentin Formation

The original dentin formation during tooth development exhibits us the biological process which we expect to work during dentin regeneration. Tooth development is initiated by the interaction between epithelium and mesenchyme [6]. Dentin formation starts at the interface of epithelium and mesenchyme, beginning at late bell stage [7]. In the bell stage, with the maturation of ameloblasts near the basement membrane, the inner enamel epithelium produces signaling molecules inducing dental papilla cells turning into odontoblasts. As the odontoblast maturation starts, odontoblasts' bodies are elongated with one end towards the dental papilla, while the other attaching to the basement membrane, and then develop into the odontoblastic processes extending into dentinal tubule. Odontoblasts are responsible for secreting organic matrix including collagen, noncollagenous protein, proteoglycan, and glycoprotein. When minerals complete deposition in the dentin matrix, dentin proceeds to mineralize and is ultimately matured. The mature dentin protecting the pulp with enamel lying in the outer layer can be divided into three parts—dentinal tubules, odontoblastic processes, and extracellular matrix. Dentinal tubules are filled with tissue fluid and odontoblastic processes. Odontoblastic processes are very sensitive to stimuli and play crucial roles in host defense. Extracellular matrix comprises peritubular dentin, intertubular dentin, interglobular dentin, Tomer's granular layer, and predentin [8].

Upon injury, would healing process in dentin-pulp initiates. It is a pathological event reflecting the extent and severity the dentin-pulp complex has suffered, and alongside the tertiary dentin formation, reactionary, and reparative dentinogenesis included. When the injury is mild enough for primary odontoblasts to survive, the following formed dentin can contain a completely regular tubular structure, which is defined as reactionary dentin. However, more intense injury strikes the dentin-pulp complex causing local death of the primary odontoblasts at the injury site and triggering a cascade of progenitors, most possibly dental pulp stem cells, recruited to the specific site, and ultimately forming reparative dentin with atubular morphology.

Zooming in to the cellular level, following tooth injury, cell death and cell replacement take place. In recreationary dentinogenesis, primary odontoblasts survive and, upon mild stimuli, keep secreting regular tubular dentin. While in reparative dentinogenesis, primary odontoblast death is followed by new generation of odontoblast-like cells, lining the pulp interface with dentin and secreting atubular dentin-like matrix. However, lacking unique molecular or morphological markers and unclear morphological evidence makes the identification of odontoblast-like cells and its origination difficult [9]. Several mesenchymal stem cell (MSC) populations, largely dental pulp stem cells, and fibroblasts are reported to contributing to the odontoblast-like cell formation, but evidence is limited.

Regenerative endodontics including vital pulp therapy, pulp revitalization, and cell homing treatments aims at healing the pulp injury with natural manners [10]. Bonafide bioactive pulp-dentin tissue is the desired result. Yet, due to limitations of current technique and materials, activation of wound healing responses to preserve the rest alive dentin-pulp complex with tertiary dentin and odontoblast-like cells formation has been taken as an interim step. Regenerative endodontics must fully consider the ability losing of tissue repairing, cell replacing, and dentin-pulp regeneration due to aging, injury, or genetic defects to rebuild physiological function. To achieve this, efforts have been exerted on studies including differentiation and formation of odontoblast-like cells and the underlying mechanism pathways. Among all the investigated biological factors, growth factors have shown its vital and irreplaceable role in the mist.

## 3. Growth Factors in Dentin Formation

Dentin formation is a complex process involving sequential and ordered deposition of an extracellular matrix, followed by its mineralization [11]. The formation of odontoblasts or odontoblast-like cells and the dentin formation is a continuous process. Growth factors affect the whole process directly or indirectly. Up till now, multiple factors including the IGFs, TGFs, and BMPs have been proved to play an irreplaceable role in dentin formation.

BMP family regulates various biological processes, such as cell proliferation, differentiation, migration, and extracellular matrix remodeling [12]. BMP-2 and BMP-7 could serve as odontogenic and osteogenic differentiation enhancers of human tooth germ cells (hTGSCs) [13]. Deficient in BMP-2 and BMP-4 caused dentin reduction and enlarged pulp chambers during tooth development [14]. BMP-2/FGF-9 signaling and TGF-*β*/BMP signaling were proved to be vital for odontoblast maturation and dentin formation in both temporal and spatial manner [15, 16]. BMP family contributes to dentin formation by promoting odontogenic differentiation and subsequently dentin quantity.

Among TGF family, TGF-*β*s has long been proven indispensable in dentin formation [17]. TGF-*β*1 increased the mineralization and ALP activity of dental pulp cells which marked the enhancement of odontogenic differentiation by regulating transcription of two critical noncollagenous proteins, dentin sialophosphoprotein (DSPP), and dentin matrix acidic phosphoprotein 1 (DMP1), in odontoblasts [18]. TGF-*β*2 strongly upregulated when pulp cells differentiated into odontoblasts in vitro [19]. In addition, elevated TGF-*β*2 signaling in dentin resulted in sex-related enamel defects [20]. Upregulated TGF-*β*3 enhanced odontogenic differentiation and the formation of ectopic dentin [21]. TGF family act mainly on the formation and function of odontoblasts.

IGFs have also been investigated and have shown great potential in regulating dentin formation.

## 4. The IGF Axis: IGFs, IGF-1R, IGFBPs, and Postreceptor Signaling

IGF family composes of IGF-1 and IGF-2 participant in massive physiological activity, including development, growth, organogenesis, metabolism, cell proliferation, and cell differentiation [22]. IGF-1 and IGF-2 contains 70 and 67 amino acids, respectively. Both IGF-1 and IGF-2 are composed of one single polypeptide chain and four distinct domains-B, C, A, and D domains from N-terminal to C-terminal. IGF-1 has a 62% amino acid similarity with IGF-2 and 60% similarity with insulin. This is the origin of nomenclature of insulin-like growth factors [23, 24].

IGF receptors have different affinities for different ligands. Both IGF-1 and IGF-2 can act via the type 1 IGF receptor (IGF-1R), which is a dimeric transmembrane glycoprotein expressed ubiquitously both pre- and postnatally. IGF-1R contains two separate IGF-contacting sites, the high affinity-binding site made up with L1 and *α*CT domains and the low affinity-binding site composed of the Fn-III-1 domain. The type 2 IGF receptor (IGF-2R) is a single-chain type I intramembrane protein, which entraps the extracellular IGF-2 exclusively.

IGF-binding proteins (IGFBPs) are a group of proteins who regulate IGF ligand function. IGFBP1-6 share 50% homology and have high affinities for IGF-1 and IGF-2. 80% of the total IGF-1 are bound by IGFBP-3, who is the most important subtypes of IGFBPs for IGF-1, while IGFBP-6 is essentially IGF-2 specific. Most IGFBPs compete for activity of IGFs at the receptor level and antagonize IGF function, while some (e.g., IGFBP2) appear to amplify IGF signaling. IGFBP7-10 also play a role in IGF axis. They share conserved N-terminal domain of the conventional IGFBPs. But unlike IGFBP1-6, IGFBP7-10 show low affinity for IGFs. IGFBP7 is proved to be at least 5-fold to 25-fold lower than conventional IGFBP1-6 in binding IGFs with its IGF-dependent and IGF-independent cell growth regulating function [25, 26]. All these IGFBPs have been proven to exert irreplaceable effects on IGF ligand-receptor binding process and to be thus essential in postbinding cell events. It is widely believed that tissue specific effects of IGFs were largely related with the tissue specific expression of IGFBPs [27].

Once binding to the cognate receptor, their inner kinase domain starts to autophosphorylate, leading to activation of two main downstream pathways, phosphatidylinositol 3-kinase (PI3K)-AKT/mammalian target of rapamycin (mTOR) pathway, and Ras-mitogen-activated protein kinase (RAS-MAPK) pathway. PI3K-AKT/mTOR pathway activated by insulin receptor substrate (IRS) predominantly leads to metabolic outcomes. The SHC-initiated RAS-MAPK pathway controls mitogenic outcomes, both play vital roles in the host defense of dental tissue injury, wound healing, and pulp regeneration [28, 29].

## 5. IGFs in Dentin Formation and Regeneration

IGFs have been revealed to drive cell proliferation and differentiation during the tooth development. Recent studies showed that IGF-1 had an essential role in cellular migration and cell proliferation in many dental tissues [30]. Immunohistochemical localization proved the occurrence of IGF axis in human deciduous teeth and human permanent teeth from root to enamel, implicating its key role in tooth development [31, 32]. When root development started, IGF-1 expression soared in dental pulp [33]. Fujiwara et al. proved that the presence of IGF-1 resulted in elongation of Hertwig's epithelial root sheath (HERS) and increased cell proliferation in its outer layer compared with the control. This indicates its regulatory effect on early root formation through HERS [34]. Furthermore, the IGF axis can also regulate the formation of amelogenin, ameloblastin [35], and dentin matrix through paracrine/autocrine, hence confirmed to be crucial for amelogenesis and dentinogenesis [36, 37]. IGFBP5-7 were found to be upregulated during tooth germ mineralization in vivo and to be differentially localized in ameloblasts and odontoblasts, showing their potential role in downregulating mineralization [38]. IGF axis also reaches the periodontal ligament. Konermann et al. found a temporal increase of IGF-2 and IGFBP-6 in periodontal ligament cells (PDLCs) in vivo, as opposed to a general decrease at protein level, arousing the researchers' interest for further investigation. They reported the peculiar enhancement of IGF-2 and IGFBP-6 led to the reduced differentiation and proliferation ability of PDLCs [39].

Dental papilla cells turning into odontoblasts through cell fate determination is a pivotal step for dentinogenesis, where multiple signaling pathways and factors are involved, and IGF axis has a place. Similarly, the odontogenic differentiation of stem cells originated from dental tissues is also governed by IGF axis. In stem cells from dental papilla (SCAPs), IGF-1 were investigated to bind to IGF-1R, activating phosphorylation of MAPK pathways, leading to enhance the differentiation and mineralization of SCAPs by increasing key markers' expression, including Alp, Runx2, Osx, Ocn, Col-I, Dspp, and Dmp-1. This effect could be enhanced by microRNA hsa-let-7c depression whilst reversed by the overexpression [40]. Evidence accumulates that IGF-1 also contributes to the proliferation and differentiation of dental pulp stem cells (DPSCs). Magnucki et al. studied how IGF-1, IGFBP-3, and IGF-1R expressed in STRO-1-positive DPSCs of fully impacted wisdom teeth. Protein levels of IGF-1, IGFBP-3, and IGF-1R were shown to be particularly higher in young third molars with ongoing development and especially the STRO-1-positive DPSCs. It can be speculated that IGF-1 axis governed a variety of underneath mechanism in the final period of tooth development and the pulp cell differentiation [41]. Under the high glucose conditions, DPSCs showed low proliferation and differentiation ability with reduced ALP activity and mineralization. However, adding IGF-1 can significantly reverse the effects. The reduced ability of DPSC proliferation, differentiation, and mineralization induced by high GLU could be rescued by IGF-1 [42]. Besides, IGF-2 is also involved. Khan et al. found that IGF-2 expression is active in both epithelial and mesenchymal area, in developing cusp mesenchyme, and in newly formed enamel layer and dentin tubules. Methylation of cytosine-phosphate-guanine (CpG) islands in Igf2 underwent a time-dependent increase with correlated decreased levels of DLK1 and IGF-2 proteins in the tooth germ. Thus, IGF-2 expression reduced during tooth development regulated by epigenetic factors [43]. By binding to IGF-1R, IGF-2 could enhance the cell mitogenic activity leading to cell proliferation. In contrast, when IGF-2 binding to IGF-2R, it was subsequently trafficked to the lysosome, where IGF-2 was degraded, and thus, the cell mitogenic activity was largely weakened [44, 45]. The SCAPs showed stronger osteo-/dentinogenic differentiation potentials with the rhIGF2 compared with the control group indicating its positive effect towards odontogenic differentiation [46].

When pulp injury happens, pathological events emerge [47, 48]. IGF axis demonstrated to play an important role in the formation of dental mineralized tissue [49]. Alkharobi et al. found that DPSCs originated from the teeth suffered from superficial caries showed higher potential to differentiate into odontoblasts and/or into osteoblasts, indicating the exposure to the mild inflammatory condition may be one positive factor. Along with the mild inflammatory, the intracellular IGF axis components of DPSCs dramatically changed with IGF-2 and IGFBP-2 upregulated and IGFBP-3 downregulated. It implicated that IGF axis-oriented mineralization might occur in DPSCs from carious teeth [50]. In vitro studies showed the cell volume mitotic index and cell differentiation of DPSCs from mouse mandibular molars were both increased by improving IGFs, suggesting IGFs' role in optimizing odontogenic differentiation of DPSCs [36, 51]. Deposition of dentin extracellular matrix could only proceed with the presence IGF-1 rather IGF-2. The expression of DSPP mRNA can be decreased both by IGF1 and IGF2, while IGF2 is more talent in it [35]. EphrinB1-EphB2 interaction has been proved to regulate odontogenic/osteogenic differentiation from dental pulp cells in vitro [52]. Matsumura et al. found that ephrinB1 was strongly expressed in odontoblasts 4 weeks postinjury in a pulp exposure model. They also found that with the inhibition of IGF-1 receptor signaling inducing the block of both Ras/Raf-1/MAPK pathway and the PI3K/Akt/mTOR pathway, specifically inhibited EphB2 expression and ephrinB1 gene expression, respectively. Hence, the IGF-1/ephrinB1 axis involves in the early period of tooth injury events [53].

With the roles of growth factors in dentin formation and regeneration getting more and more defined, proper carriers facilitating clinic application are gaining more and more attention. Exosomes, small double-lipid layer particles containing proteins, mRNA, and miRNA, can convey a multiple of bioinformation and stimuli. Exosomes secreted by MSCs showed strong regenerative potential and immunomodulation [54]. Cai et al. successfully promoted neurite outgrowth in vitro and enhanced regeneration after sciatic nerve injury in vivo by applying exosomes containing natural growth factors from adipose-derived MSCs [55]. In addition, exosomes from the human periapical cyst mesenchymal stem cells (hPCy-MSCs) were proved to be effective in the pathogenesis of Parkinson's disease [56]. For its excellent biocompatibility, biological safety, and bioeffect, exosomes might also be the ideal carrier of IGFs, waiting for scientists to further investigate.

## 6. Summary and Future Directions

**Figure 1:**
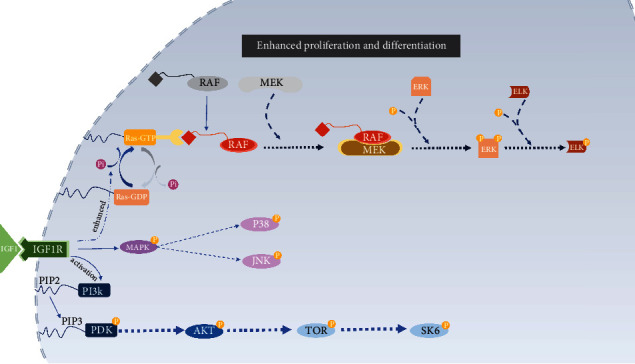
The postevent of IGF1 and IGF1R binding in cells. After IGF1 binds to IGF1R, two main pathways are activated. Ras-GTP expression is enhanced, leading to the activation RAF pathway, thus activating ELK and MAPK pathway. PI3K is activated, then boost AKT and mTOR expression.

The underneath mechanism of dental wound healing and repair sheds light on advanced healing strategies inducing pulp regeneration processes. IGFs orchestrating crucial cell events in dentin formation makes it one of the potential candidates for dentin regeneration. Yet, there are still gaps between our current knowledge about dentin formation and what is really going to there. Previous research proved spatio-temporal expression of IGF axis in dentin development, arousing interests in probing into the role of IGFs in tooth development. Further research revealed IGF axis' involvement in dentin and dentin-like tissue formation. Additionally, Ras/Raf-1/MAPK and PI3K/AKT/mTOR pathways were found activated by IGF-axis during this biological process. Yet, the adequate release and spatio-temporal expression of IGF-axis in an active form and the manual control release of IGF-axis imitating bioprocess have not yet been investigated. The gaps mentioned above also reminds us of potential barriers within the IGFs' application in the future. Moving forward, it will be critical to further investigate growth factors both at the genomics level in biologically relevant cell types and in the potential therapeutic application (Figure 1).

## Data Availability

Data sharing is not applicable.
